# β-Carboline Alkaloids from *Peganum harmala* Inhibit *Fusarium oxysporum* from *Codonopsis radix* through Damaging the Cell Membrane and Inducing ROS Accumulation

**DOI:** 10.3390/pathogens11111341

**Published:** 2022-11-13

**Authors:** Zihao Zhu, Shujuan Zhao, Changhong Wang

**Affiliations:** 1 The SATCM Key Laboratory for New Resources & Quality Evaluation of Chinese Medicine, Institute of Chinese Materia Medica, Shanghai University of Traditional Chinese Medicine, Shanghai 201203, China; 2 The MOE Key Laboratory for Standardization of Chinese Medicines, Shanghai Key Laboratory of Compound Chinese Medicines, Shanghai University of Traditional Chinese Medicine, Shanghai 201203, China

**Keywords:** *Fusarium oxysporum*, antifungal, *Peganum harmala*, β-carboline alkaloids, harmane, transcriptome

## Abstract

*Fusarium oxysporum* is a widely distributed soil-borne pathogenic fungus that can cause medicinal herbs and crops to wither or die, resulting in great losses and threat to public health. Due to the emergence of drug-resistance and the decline of the efficacy of antifungal pesticides, there is an urgent need for safe, environmentally friendly, and effective fungicides to control this fungus. Plant-derived natural products are such potential pesticides. Extracts from seeds of *Peganum harmala* have shown antifungal effects on *F. oxysporum* but their antifungal mechanism is unclear. In vitro antifungal experiments showed that the total alkaloids extract and all five β-carboline alkaloids (βCs), harmine, harmaline, harmane, harmalol, and harmol, from *P. harmala* seeds inhibited the growth of *F. oxysporum*. Among these βCs, harmane had the best antifungal activity with IC_50_ of 0.050 mg/mL and MIC of 40 μg/mL. Scanning electron microscopy (SEM) and transmission electron microscopy (TEM) results revealed that the mycelia and spores of *F. oxysporum* were morphologically deformed and the integrity of cell membranes was disrupted after exposure to harmane. In addition, fluorescence microscopy results suggested that harmane induced the accumulation of ROS and increased the cell death rate. Transcriptomic analysis showed that the most differentially expressed genes (DEGs) of *F. oxysporum* treated with harmane were enriched in catalytic activity, integral component of membrane, intrinsic component of membrane, and peroxisome, indicating that harmane inhibits *F. oxysporum* growth possibly through damaging cell membrane and ROS accumulation via regulating steroid biosynthesis and the peroxisome pathway. The findings provide useful insights into the molecular mechanisms of βCs of *P. harmala* seeds against *F. oxysporum* and a reference for understanding the application of βCs against *F. oxysporum* in medicinal herbs and crops.

## 1. Introduction

High-quality medicinal herbs are the material basis for the inheritance and development of traditional Chinese medicine and are strategic resources related to the national economy and people’s livelihood. In recent years, with the growing demand for high-quality medicinal herbs at home and abroad, the species and area of artificially cultivated medicinal herbs have increased significantly [1]. However, problems such as root rot and fusarium wilt are becoming more and more serious in the process of planting medicinal herbs, such as *Panax ginseng* [2], *Codonopsis radix* [3], *Panax quinquefolius* [4], and *Crocus sativus* [5], resulting in the decline of yield and quality of medicinal herbs [6]. *Fusarium oxysporum*, a widely distributed soil-borne pathogenic fungus with strong destructiveness, is the main pathogen causing root rot or fusarium wilt of the medicinal plants [7]. It can infect more than 150 crops, such as banana, tomato, soybean, and wheat before harvest [8,9,10], and it was listed as one of the top ten plant pathogenic fungi in the world in 2012 [11]. A recent prediction showed that the banana wilt caused by *F. oxysporum* worldwide would cause economic losses of more than 10 billion dollars by 2040 [12]. 

In addition, *F. oxysporum* can produce some secondary metabolites in the process of infection of crops, such as fusaric acid, fumonisins, and beauvericin [13,14]. These toxins may cause nausea, diarrhea, dizziness, fever and food-poisoning leukopenia, which pose a potential threat to livestock [15] and human health [16].

At present, chemical antimicrobial agents, such as azoxystrobin and thiophanate methyl, are often used to prevent and treat plant diseases caused by agriculture fungal pollution [17,18]. Azoxystrobin is a broad-spectrum fungicide with good activity against almost all fungal diseases and it is the best-selling fungicide in the world. However, long-term heavy use of such chemicals would cause drug resistance of pathogenic fungi, pollutes the environment, and has potential food safety risks, threatening human health, which does not meet the needs of the sustainable development of modern agriculture [19]. Therefore, it is urgent to develop green, safe and effective natural antimicrobial agents to control soil-borne diseases caused by *F. oxysporum*.

**Figure 1:**
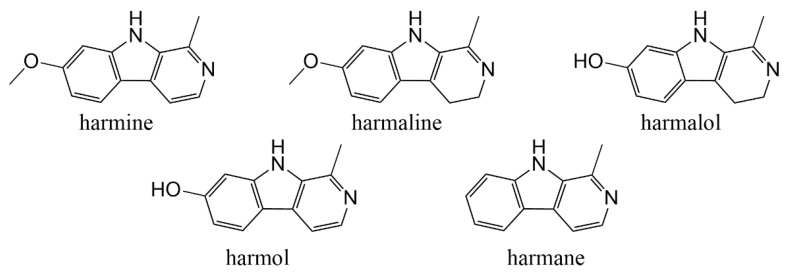
Structures of the five main βCs in *P. harmala* seeds.

Many natural plant active compounds have attracted much attention due to their excellent antimicrobial activities, such as chlorogenic acid [20], allicin [21], eugenol [22], and curcumin [23]. *Peganum harmala*, a perennial herb from the Zygophyllaceae family, is widely distributed in arid grasslands in desert areas, lightly salinized sandy land on the edge of oasis, loamy low hillsides or river valley dunes of Central Asia, Europe, and southern South America. It is commonly used in folk medicine to treat fever, cough, diarrhea, hypertension, asthma, jaundice, and skin diseases [24]. It is rich in β-carboline alkaloids (βCs), the content of which in seeds reaches up to 10%, including harmine, harmaline, harmalol, harmol, and harmane (Figure 1) [25,26,27]. Studies have shown that extracts from seeds of *P. harmala* have broad spectrum activities against fungi, such as *F. oxysporum*, *Aspergillus niger*, *Cryptococcus neoformans*, *Alternaria* sp., and *Epidermophyton floccosum* [27,28]. However, studies on the antifungal activity of βCs from *P. harmala* against *F. oxysporum* are limited and the antifungal mechanism has not been elucidated.

In this paper, the potential antifungal effect of βCs, especially harmane on *F. oxysporum* was investigated. Scanning electron microscopy (SEM), transmission electron microscopy (TEM), and transcriptome analysis were conducted to explore the inhibition mechanism, which showed that harmane inhibits the mycelial growth of *F. oxysporum* possibly through regulating the expression of genes related to steroid biosynthesis and peroxisome metabolism. This study provides a reference for understanding the application of βCs in medicinal herbs and crops.

## 2. Materials and Methods

### 2.1. Isolation and Identification of F. oxysporum 

*F. oxysporum* was isolated according to the previously reported methods [29]. Briefly, fungal pathogens were isolated from root of *Codonopsis radix* with root rot collected in Gansu province of China, and grown on potato dextrose agar (PDA). After 5 days of culturing, the colony was convex flocculent, pinkish white, slightly purple. The mycelium was white and dense. It was identified as *F. oxysporum* by morphological characteristics and 16S rRNA sequence analysis (Genbank MK966308). 

Spore suspension was prepared according to the literature with slight modifications [30]. In short, the spore suspension was collected by flooding the surface of the 7-day-old culture plates with sterile water and filtering with sterile degreasing cotton. Then, the *F. oxysporum* spore suspension was diluted to a concentration of approximately 1.0 × 10^6^ CFU/mL, using a hemocytometer.

### 2.2. Chemicals 

Harmine (CAS NO. 442-51-3, purity 98%), harmaline (CAS NO. 304-21-2, purity 98%), harmalol (CAS NO. 525-57-5, purity 98%), harmol (CAS NO. 487-03-6, purity 98%), and harmane (CAS NO. 486-84-0, purity 98%), and total alkaloid extracts were isolated from *P. harmala* seeds by our laboratory [31]. The content of harmine and harmaline in total alkaloid extracts was 55.3%. The structures of βCs included in the project are shown in Figure 1. Azoxystrobin (CAS NO. 131860-33-8, purity 98%) was purchased from Beijing Norma Standard Technology Co., Ltd (Beijing, China). 

### 2.3. Inhibition of Total Alkaloids on Mycelial Growth 

The inhibition effect of total alkaloid extracts from *P. harmala* seeds against *F. oxysporum* were tested by agar diffusion method [32]. Alkaloid extracts from *P. harmala* were mixed with PDA, and the final concentrations were 0.05, 0.1, 0.2, 0.4, and 0.5 mg/mL. Azoxystrobin at dose of 0.4 mg/mL was used as a positive control. *F. oxysporum* was inoculated on the PDA and cultured at 28 °C for 5 days. PDA without alkaloid was used as a control. The mycelial growth diameter of *F. oxysporum* colony was measured and the inhibition rate was calculated according to the following Formula (1).

(1) $$\mathrm{Inhibition}\;\mathrm{rate}\;(\%)=\frac{\mathrm{the}\;\mathrm{diameter}\;\mathrm{of}\;\mathrm{control}\;-\;\mathrm{the}\;\mathrm{diameter}\;\mathrm{of}\;\mathrm{treatment}}{\mathrm{the}\;\mathrm{diameter}\;\mathrm{of}\;\mathrm{control}}\times 100\%$$

### 2.4. Inhibition of Five βCs on Mycelial Growth and IC_50_ CALCULATION 

The inhibition effect of the five βCs on *F. oxysporum* was tested in the same way as total βCs. The IC_50_ was analyzed using SPSS (version 25.0, Norman H. Nie, C. Hadlai (Tex) Hull and Dale H. Bent, CA, USA).

### 2.5. Determination of Minimal Inhibitory Concentration (MIC)

According to the American Society for Clinical and Laboratory Standards (CLSI) standard, the MIC of βCs against *F. oxysporum* was determined by tube double dilution method in a 96-well plate [33]. βCs were separately mixed with Potato Dextrose Broth (PDB) in the concentration range of 0.625–50 μg/mL, and 4 mL of the mixed solution was added into 20 μL of the conidial suspension. Then, each concentration of the mixed solution was successively distributed to three wells of the 96-well plate. PDB without βCs was used as the control group. The MIC was defined as the lowest drug concentrations that caused complete visible inhibition of growth.

### 2.6. Scanning Electron Microscopy (SEM) 

The morphology of *F. oxysporum* after harmane treatment was observed with SEM according to the literature [34]. The spore suspension was added into PDB and cultured at 28 °C (120 rpm) for 48 h. After centrifugation at 4000× *g* for 5 min, the mycelium was suspended again in PBS (pH 7.2). The βCs were added to the buffer solution to make the concentration MIC and incubated at 28 °C for 12 h, with anhydrous ethanol as the control group [35].

The samples were fixed in 2.5% glutaraldehyde, washed with PBS three times, 15 min each time, fixed with 1% osmic acid solution for 1 h, washed three times, 15 min each time. The samples were dehydrated with ethanol solution of five concentration gradients (including 30%, 50%, 70%, 80%, 90% and 95%). Each concentration was treated for 15 min, and then 100% ethanol was used twice, 20 min each time. The sample was treated with the mixture of ethanol and isoamyl acetate for 30 min, and then treated with pure isoamyl acetate for 1 h, dried, coated and examined by SEM (×10.0K and ×20.0K, U8010, Hitachi, Tokyo, Japan).

### 2.7. Transmission Electron Microscopy (TEM) 

For TEM, mycelia were treated the same way as SEM and slightly modified. In short, the treated samples were fixed in 2.5% glutaraldehyde and washed three times with PBS for 15 min each time. The samples were dehydrated with ethanol solution of five concentration gradients (including 30%, 50%, 70%, 80%, 90% and 95%). Each concentration of the sample was treated for 15 min and then treated twice with 100% ethanol for 20 min each time. The samples were embedded for 3 h and sliced in an ultra-thin cutting machine (UC7, Leica, Wetzlar, Germany). The samples were stained with lead citrate solution and 50% ethanol saturated solution of uranium dioxide acetate for 5 min, respectively, and then examined by TEM (H-7650, Hitachi).

### 2.8. Evaluation of Release of Cell Components 

The release of cell components was evaluated using OD_260_ determined with UV spectrophotometry [35]. To do that, the 1 × 10^6^ CFU/mL suspension was mixed with PDB and cultured at 28 °C (120 rpm) for 48 h. After centrifugation at 4000× *g* for 15 min, the mycelia were collected and washed with sterile water three times. Then, the mycelia were suspended in phosphate buffer solution (PBS, pH 7.2), supplied with harmane at the final concentration of 0.5 MIC and MIC, then incubated at 28 °C for 4 h, 8 h, and 12 h, respectively. Samples were centrifuged at 4000× *g* for 5 min to collect supernatant for OD_260_ measurement. PBS (pH 7.2) was used as the control.

### 2.9. Measurement of Electrical Conductivity 

The influence of harmane on electrical conductivity of *F. oxysporum* was measured according to the literature [32]. The sample was treated in the same way as for cell component assay. The conductivity of the supernatant of different samples was determined using conductivity meter (DDS-11D, JingKe, Shanghai, China). 

### 2.10. ROS Assay 

The content of ROS in cells was evaluated by Reactive Oxygen Species assay kit (Beyotime, Shanghai) combined with fluorescence microscopy. The method of culture and treatment of samples was described in SEM. The DFCH-DA probe was added into the treated samples and incubated at 37 °C for 30 min. After centrifugation, the supernatant was washed twice with PBS, and the precipitation was collected and observed under bright light and green light by fluorescence microscopy (×10, Olympus IX81, Tokyo, Japan).

### 2.11. Annexin V-FITC/PI Double Staining Assay 

The cell death rate was analyzed using Annexin V-FITC Apoptosis detection kit (Beyotime, Shanghai, China) combined with fluorescence microscopy, which could also discriminate types of cell death (apoptotic or necrotic cell death) [30]. The method of culture and treatment of samples was described in SEM. Briefly, a total of 500 μL of the treated sample was mixed with 5 μL of Annexin V-FITC and then 5 μL of propidium iodide (PI) was added, incubated at 25 °C for 10 min, and imaged under fluorescence microscopy (Olympus IX81).

### 2.12. Transcriptomic Analysis 

The total RNA of the treated samples was extracted with TRIzol® Reagent (Invitrogen, Carlsbad, CA, USA), according the manufacturer’s instructions, and genomic DNA was removed using DNase I (TaKara, Kyoto, Japan). Its concentration, purity and integrity were detected by Nanodrop2000 (NanoDrop Technologies, Waltham, MA, USA). The transcriptome library was prepared following Truseq^TM^ RNA sample preparation kit from Illumina (San Diego, CA, USA) using 1 μg of total RNA. Then, the synthesized cDNA was subjected to end-repair, phosphorylation and ‘A’ base addition according to Illumina’s library construction protocol. Libraries were size selected for cDNA target fragments of 300 bp on 2% Low Range Ultra Agarose followed by PCR amplified using Phusion DNA polymerase (NEB) for 15 PCR cycles. After quantified by TBS380, paired-end RNA-seq sequencing library was sequenced with the Illumina NovaSeq 6000 sequencer (2 × 150 bp read length). The original sequencing data was subjected to quality control using SeqPrep (https://github.com/jstjohn/SeqPrep, accessed on 15 November 2021) and Sickle (https://github.com/najoshi/sickle, accessed on 15 November 2021) software to obtain clean data. These clean data were compared with the reference genome (Fusarium_oxysporum, http://fungi.ensembl.org/Fusarium_oxysporum/Info/Index, accessed on 15 November 2021) using HiSat2 (http://ccb.jhu.edu/software/hisat2/index.shtml, accessed on 15 November 2021) to obtain mapped data for subsequent transcript assembly, expression amount calculation, and others. The RSEM (http://deweylab.biostat.wisc.edu/rsem/, accessed on 22 November 2021) software was used to perform progressive analysis on the expression levels of genes and transcripts to obtain read counts, and DESeq2 (http://bioconductor.org/packages/stats/bioc/DESeq2/, accessed on 22 November 2021) software was used to identify differentially expressed genes (DEGs) between samples using FDR < 0.05 & |log2FC| ≧ 1 as the standard. DEGs were annotated and analyzed for enrichment in the GO database (http://www.geneontology.org, accessed on 3 July 2022) and the KEGG database (http://www.genome.jp/kegg/, accessed on 3 July 2022), respectively.

### 2.13. Statistical Analysis 

Three independent experiments were performed for each assay. All statistical analyses were performed using GraphPad Prism 9.0.0 (Harvey Moltusky, San Diego, CA, USA), and regression analysis was used to determine the significant differences with 95% confidence (*p* < 0.05).

## 3. Results

### 3.1. Inhibition of Total Alkaloid Extracts from P. harmala on Mycelial Growth 

**Figure 2:**
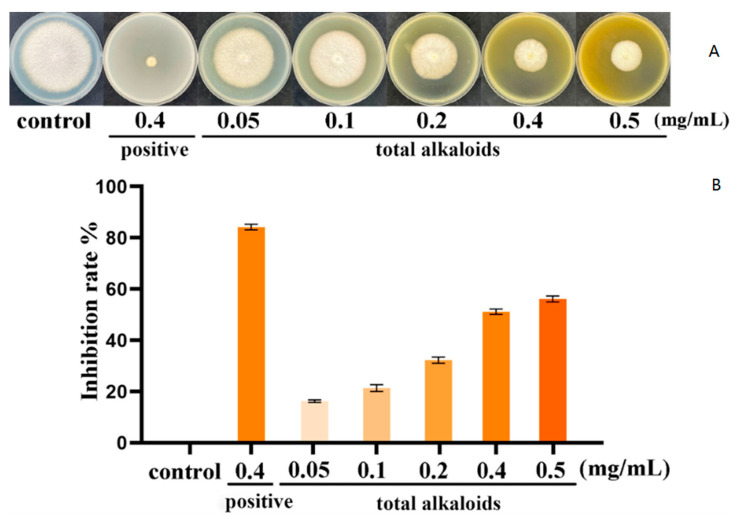
The inhibitory effects of total alkaloid extracts from *P. harmala* against *F. oxysporum*. (**A**) The inhibitory effects of total alkaloids on mycelial growth of *F. oxysporum*. (**B**) The inhibition rate of total alkaloids against *F. oxysporum*.

Results revealed that total alkaloids exhibited inhibition on mycelial growth (Figure 2A). The inhibitory effect of total alkaloids on mycelial growth was concentration-dependent. The mycelial growth inhibition rates at concentrations of 0.05, 0.1, 0.2, 0.4, and 0.5 mg/mL were 16.3%, 21.4%, 32.2%, 51.3% and 56.3%, respectively (Figure 2B). The mycelial growth inhibition rate of the positive control group at dose of 0.4 mg/mL was 84.2%. These results showed that total alkaloid extracts from *P. harmala* can inhibit the growth of *F. oxysporum*.

### 3.2. Inhibition of Five Target βCs on Mycelial Growth 

**Figure 3:**
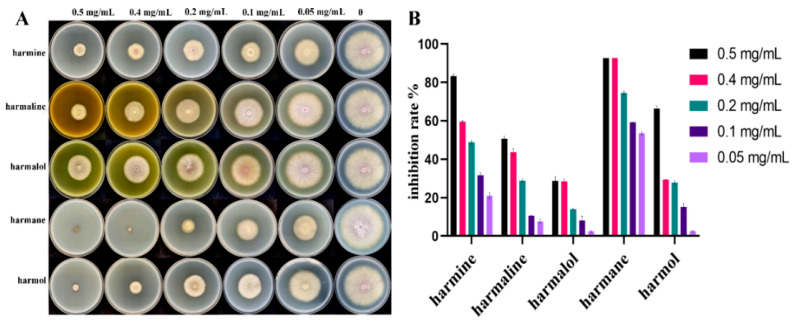
The inhibitory effects of the five βCs on *F. oxysporum*. (**A**) The inhibitory effect of the five βCs on mycelial growth of *F. oxysporum*. (**B**) The inhibition rate of five βCs against *F. oxysporum*.

To further explore the effect of total alkaloids, five main alkaloids were cultured with *F. oxysporum*. As shown in Figure 3A, all the five βCs had obvious inhibitory effect on *F. oxysporum* and the inhibition zone increased with the concentration of βCs from 0.05 to 0.5 mg/mL, indicating that the antifungal effect of βCs against *F. oxysporum* was in a concentration-dependent manner. Among the five βCs, harmane had the most significant inhibitory effect. When the concentration was 0.5 mg/mL, the mycelia nearly stopped growing, and the inhibitory rate reached 100% (Figure 3B). 

The IC_50_ of the five βCs from low to high were 0.050 mg/mL (harmane), 0.143 mg/mL (harmine), 0.161 mg/mL (harmol), 0.331 mg/mL (harmaline), and 0.798 mg/mL (harmalol) (Table 1). Harmane showed the best antifungal activity and was investigated in subsequent experiments.

### 3.3. MIC 

**Figure 4:**
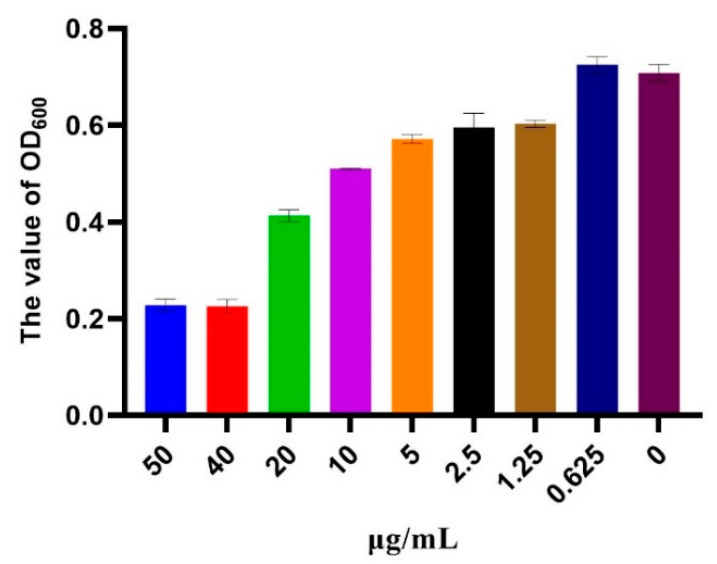
The value of OD_600_ of *F. oxysporum* cultures with different concentrations of harmane.

By observing the clarification of different concentrations, we found that when the concentration of harmane was 40 μg/mL, the fungal liquid was clear, and when the concentration was 20 μg/mL and lower, the fungal liquid was turbid. OD_600_ values are shown in Figure 4. It was determined that the MIC of harmane was 40 μg/mL.

### 3.4. SEM 

**Figure 5:**
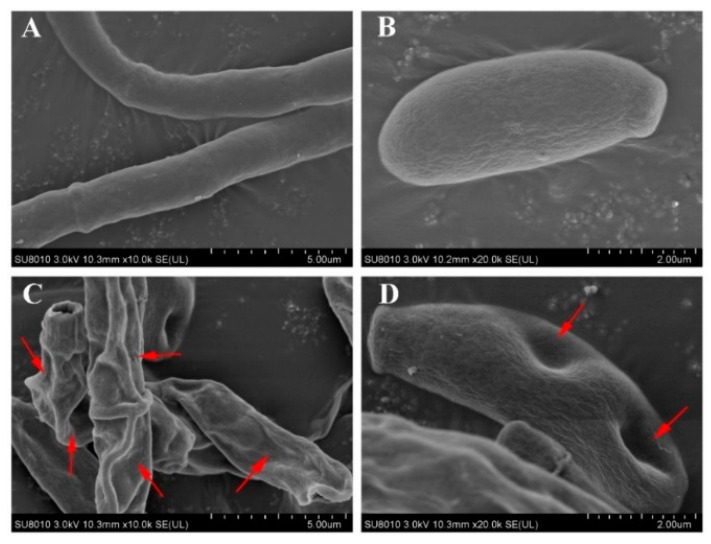
Morphology of *F. oxysporum* under SEM, (**A**,**B**) Morphology of normal growth of mycelia and spores in the control group, (**C**,**D**) Morphology of mycelia and spores induced by harmane. (**A**,**C**) ×10K, bar = 5.00 μm, (**B**,**D**) ×20K, bar = 5.00 μm.

The results of SEM analyses of *F. oxysporum* spores are shown in Figure 5. It can be observed that the morphology of hyphae and spores had undergone significant changes. From the control group, it can be seen that mycelia and spores are with a smooth surface and plump in shape, with no wrinkles and have a normal growth (Figure 5A,B). The surface of mycelia and spores in the treatment group was wrinkled, depressed, shriveled, and deformed where the red arrows pointed (Figure 5C,D). It can be seen that inhibition of harmane against *F. oxysporum* mainly affects cell morphology and leads to cell atrophy.

### 3.5. TEM 

**Figure 6:**
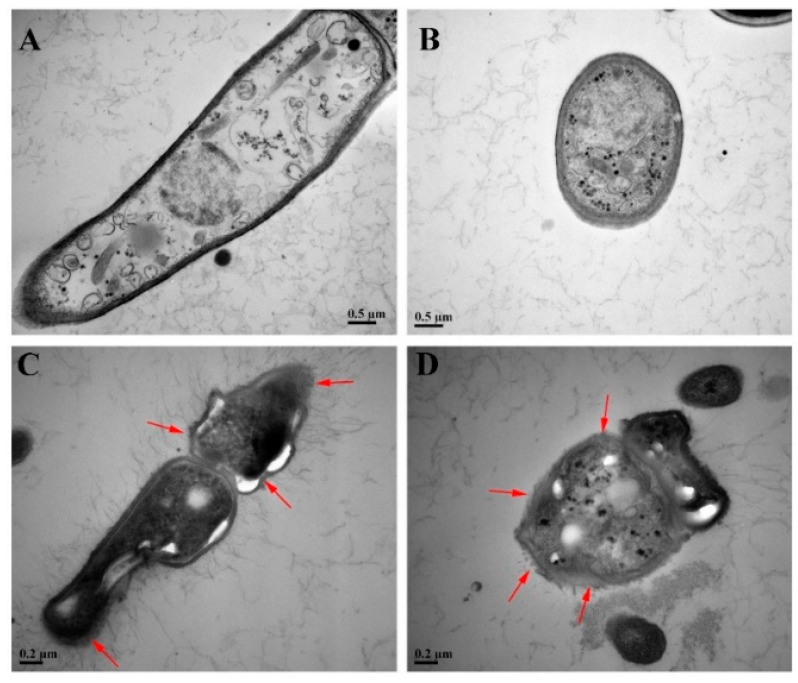
Ultrastructure of mycelia and spores under TEM, (**A**,**B**) Ultrastructure of mycelia and spores in the control group, (**C**,**D**) Ultrastructure of mycelia and spores induced by harmane. (**A**,**C**) Longitudinal section through the mycelia, (**B**,**D**) Tangential section through the mycelia. (**A**,**B**) ×25K, bar = 0.5 μm, (**C**,**D**) ×50K, bar = 0.2 μm.

The ultrastructural changes of *F. oxysporum* were further observed by TEM and results are shown in Figure 6. In the control group, the cell boundary was clear, the cell wall was complete, the thickness was uniform, the cell morphology was elliptical, the organelles were arranged neatly, and the cell growth was normal (Figure 6A,B). The mycelia in the treatment group were dissolved in irregular oval shape, the integrity of cell wall was destroyed, and the cytoplasm was blurred where the red arrows pointed (Figure 6C,D). This result confirmed that the permeability or integrity of cell membrane was destroyed.

### 3.6. Detection of Release of Cell Components and Electrical Conductivity 

**Figure 7:**
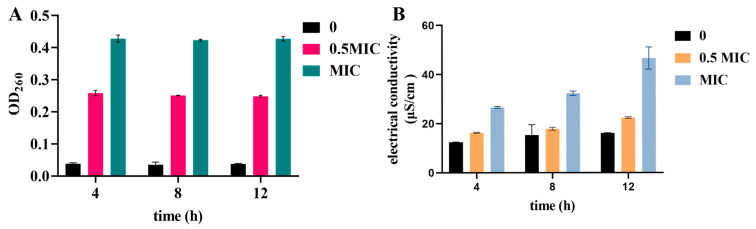
The effect of harmane on cellular component release and electrical conductivity of *F. oxysporum*, (**A**) Influence of harmane on OD_260_, (**B**) Influence of harmane on electrical conductivity.

As shown in Figure 7, at the concentrations of 0, 0.5 MIC, and MIC, harmane significantly increased the release of cell components of *F. oxysporum*. The OD_260_ was 0.43 at the concentration of MIC after incubation for 12 h (Figure 7A), which was significantly higher than that in the control group (*p* < 0.05).

With the increase of processing time, the electrical conductivity also showed an increasing trend (Figure 7B). After 12 h, the electrical conductivity of the control group was the lowest (16.13 μS/cm), and the electrical conductivity of the MIC was highest (46.6 μS/cm) compared with that of the control, with significant differences (*p* < 0.05), indicating that harmane possibly disrupted the cell membrane of *F. oxysporum* and increased its permeability.

### 3.7. Harmane Induced Accumulation of ROS 

**Figure 8:**
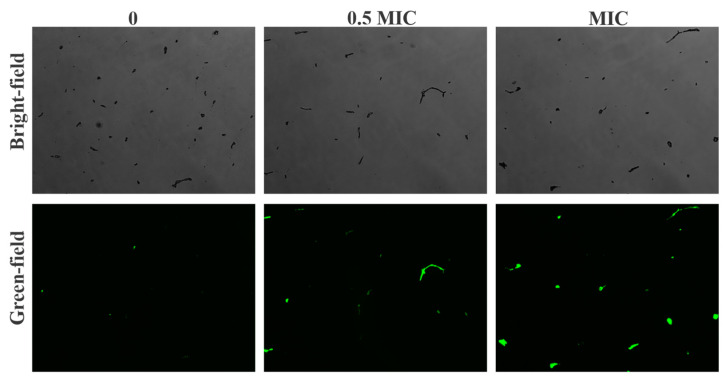
Harmane induced intracellular accumulation of ROS in *F. oxysporum*. Bright-field was the results of DCFH-DA staining of *F. oxysporum* under bright light (×10). Green-field was the results of DCFH-DA staining of *F. oxysporum* under green light (×10).

DCHF-DA staining was used to evaluate the content of ROS levels in the cells after incubation with harmane. According to the literature [36], the green fluorescence brightness is positively correlated with the content of ROS in the cell. In the control group (CK), few spores with weak fluorescence were found. When the concentration of harmane was MIC, induced intracellular accumulation of ROS was noticed. The proportion of spores producing fluorescence increased in a concentration-dependent manner after treatment of harmane (Figure 8). These results suggested that harmane could cause outbreak of ROS in *F. oxysporum*.

### 3.8. Cell Death Analysis 

**Figure 9:**
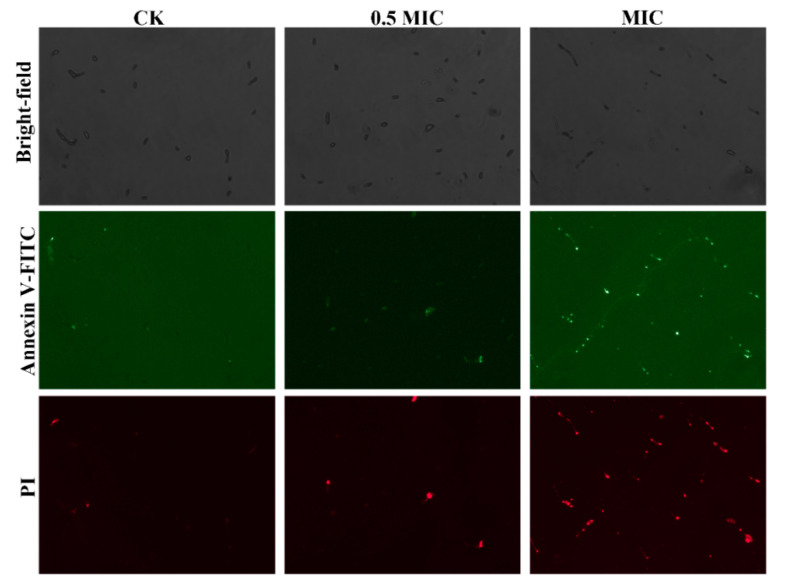
Harmane induced cell death of *F. oxysporum*. Annexin V-FITC was the results of Annexin V-FITC staining of *F. oxysporum* under green light (×10); PI was the results of PI staining of *F. oxysporum* under red light (×10).

The antifungal mechanism of harmane against *F. oxysporum* was investigated using Annexin V-FITC/PI double staining. As shown in Figure 9, after Annexin V-FITC/PI staining, spores in the control group (CK) rarely show green or red fluorescence with weak fluorescence intensity. With the increase of harmane content, the green and red fluorescence intensity and percentage of the cells were higher. Most cells in the MIC group showed fluorescence, indicating that the membrane permeability of *F. oxysporum* was damaged, leading to cell death.

### 3.9. Effect of Harmane on the Transcriptome of F. oxysporum 

Transcriptome sequencing was performed to further reveal the antifungal mechanism of harmane. We collected differently treated mycelia (0, MIC) for RNA sequencing. Principal component analysis showed that the repetitions of each sample clustered together, while different groups were separated at PC1 and PC2 levels. There were significant differences in gene expression between the two groups after treatment of alkaloid. These data demonstrated that the accuracy and reliability of RNA-sequencing for later analysis. Through the analysis of the DEGs of the two groups, a total of 8624 identical genes were obtained between the control and MIC groups. A total of 300 genes were specific to the control group, and 630 genes were specific to the harmane group. After treatment of harmane, 1883 genes were differentially expressed of which 1137 genes were up-regulated and 746 genes were down-regulated. To analyze the specific differences caused by harmane, DEGs were classified according to molecular function, biological process and cellular component in GO database. Eight terms in cellular component and six terms in biological process and molecular function were affected in *F. oxysporum* under harmane treatment. Among the terms, “membrane part”, “metabolic process” and “catalytic activity” were most significantly enriched in these three categories, respectively. 

**Figure 10:**
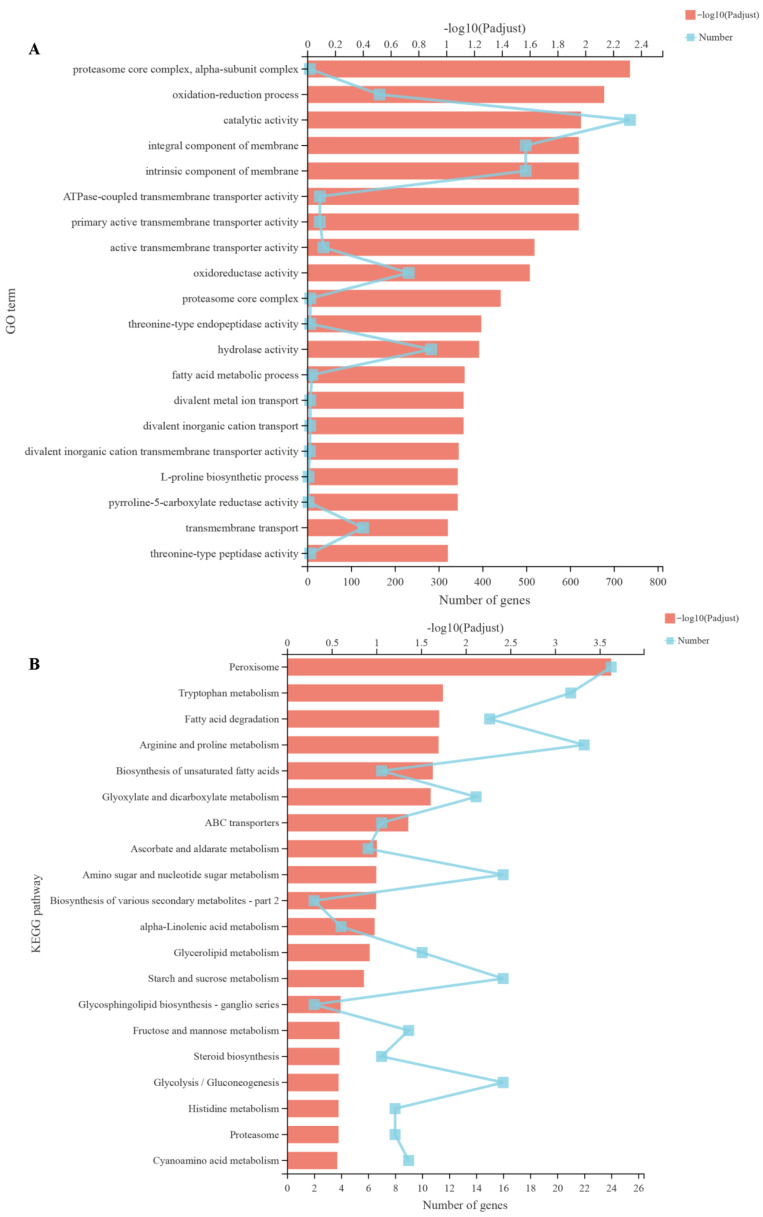
Cluster analysis and enrichment analysis in GO and KEGG databases, (**A**,**B**) Enrichment analysis of DEGs in GO and KEGG databases.

Similar to the GO annotation analysis, the GO term enrichment analysis showed that DEGs related to catalytic activity, integral component of membrane and intrinsic component of membrane were the most enriched pathways (Figure 10A) in which a unigene encoding C-5 sterol desaturase (ERG3) was significantly down-regulated. 

KEGG pathway enrichment analysis showed that the DEGs belonged to peroxisome pathway were the most enriched (Figure 10B) in which unigenes encoding peroxisomal catalase (CAT) and superoxide dismutase (SOD) were significantly decreased after harmane treatment.

## 4. Discussion

Over the years, the long-term heavy use of pesticides has made the development of new natural antimicrobial agents with good antifungal effect more and more popular [37]. *P. harmala* is a drought tolerant plant that is widely distributed in the world [24]. Extracts from seeds of this plant have antimicrobial effects on a variety of fungi, bacteria, and viruses [27]. However, there are few in-depth studies on the antifungal activity and mechanism of the total βCs or the five β-carboline alkaloids against *F.oxysporum*. In this study, the antifungal effect of βCs from *P. harmala* seed extract and the mechanism of harmane against *F. oxysporum* was investigated in order to provide evidence for the development of new, green agents against *F. oxysporum*.

The mycelial growth test of the total alkaloids showed that total alkaloids had an obvious inhibitory effect on mycelial growth. This indicated that the total alkaloids were the antifungal components in the extract of *P. harmala* seed. The results of the further mycelial growth inhibition test of five βCs showed that these βCs from *P. harmala* extract had different degrees of inhibition on *F. oxysporum*, and harmane showed the strongest antifungal activity, with IC_50_ of 0.050 mg/mL, which was lower than that of mancozeb, hymexazol and palmatine [38,39]. The double dilution method is commonly used to measure IC_50_ in general. The inhibition rates of harmine, harmaline and harmol were with significant difference at 0.4 mg/mL and 0.5 mg/mL. Yet, there was no difference of harmane at 0.4 mg/mL and 0.5 mg/mL of which the inhibition rate was 100%. In overall consideration, we made a slight modification of tube double dilution method and chose 0.5 mg/mL for the maximum concentration.

Azoxystrobin is often used as a pesticide to prevent root rot of *C. radix* in agriculture. It is a commonly used as a positive control in the study of inhibiting *F. oxysporum* [40]. At the concentration of 0.4 mg/mL, the antifungal effect of harmane is better than that of azoxystrobin, and harmane has the potential to be developed into an antifungal drug.

The MIC of harmane was 40 μg/mL, comparable to that of amphotericin B [41]. Harmane has the potential to be developed as a drug against *F. oxysporum*. At the same time, it is necessary to study the antifungal spectrum, which will be conducive to the development of broad-spectrum antifungal drugs. These results indicated that harmane had good antifungal potential and could be used as a potential fungicide against *F. oxysporum* in the future.

SEM and TEM results showed that after harmane treatment, the boundary of *F. oxysporum* cells was blurred; the cell membrane and cell wall are dissolved or even ruptured in some places, and the cytoplasm is disordered. It was proved that harmane damaged the cell membrane integrity of *F. oxysporum*. The increased permeability, the released cell components, and the increased extracellular electrical conductivity also supported this point.

There was no significant difference of OD_260_ at 4 h, 8 h, and 12 h, indicating that the intracellular nucleic acid was released within 4 h. The electrical conductivity was with significantly difference at 4 h, 8 h, and 12 h, indicating that the release process of a large number of sugars, proteins, nucleic acids, inorganic salts and other contents in the cells was relatively slow. Within 12 hours, their leakage increased linearly with time. This trend was consistent with previous reports [42]. OD_260_ and electrical conductivity have been proved to be important indicators of cell membrane damage [35]. Previous studies have proved that the butan-1-ol extract of *P. harmala* seeds could cause cell membrane damage [43].

βCs could induce accumulation of ROS in plant pathogenic fungi (*Penicillium digitatum* and *Botrytis cinerea*) [44]. The fluorescence microscopy results in this study also demonstrated that harmane induced ROS accumulation in *F. oxysporum*. High concentrations of ROS can slow down cell growth and even lead to cell death through cellular oxidative stress [45,46]. Thus, the cell death detected by Annexin V-FITC/PI staining after harmane treatment was possibly partially resulted from the accumulation of ROS. 

Further transcriptomic analysis revealed that harmane down-regulated the expression level of ERG3, CAT and SOD in *F. oxysporum*. ERG3, a key enzyme in the biosynthesis of ergosterol is involved in steroid biosynthesis [47]. The disruption of ergosterol biosynthesis resulted in increased cell membrane permeability [48]. The decrease of ERG3 expression affected the growth of fungi, resulting in the inability to produce ergosterol and destruction of membrane integrity [49]. It appears that the harmane-caused damage of cell membrane of *F. oxysporum* was possibly related with the downregulation of ERG3. Cells generate ROS through a variety of pathways, which can be cleared by SOD and CAT, thereby maintaining a dynamic balance of intracellular ROS [50]. The accumulation of ROS in *F. oxysporum* caused by harmane was likely related to the reduced expression of SOD and CAT and the ROS could not be removed normally.

According to the results of cellular component release and electrical conductivity, the cell membrane damage may occur before 4 h. It would be better to verify the expression level of key unigenes earlier.

## 5. Conclusions

In summary, it was demonstrated that the alkaloid extract and βCs from *P. harmala* could inhibit the mycelial growth of *F. oxysporum*. Among these βCs, harmane had the best antifungal activity and caused damage of the morphology of mycelia and spores of *F. oxysporum*, the integrity of cell membrane, accumulation of intracellular ROS, and cell death. Combined with transcriptome analysis, harmane may disrupt the integrity of the cell membrane by regulating steroid biosynthesis and interfering with ergosterol metabolism via down-regulating genes, such as ERG3, causing cell wall dissolution and the damage of cell membrane integrity, resulting in cell death. On the other hand, harmane interferes with the metabolism of ROS by down-regulating CAT and SOD, leading to the accumulation of ROS and damage to cells, which may also cause cell death. βCs has the potential to control *F. oxysporum* pollution as an antimicrobial agent. Therefore, future research is needed to make out the anti-*F. oxysporum* effects in fields. Our results provide important insights into the potential mechanism of βCs inhibiting fungal growth, which may be helpful for future applications of *P. harmala* in planting medicinal herbs and crops.

## Figures and Tables

**Table 1:** IC_50_ of the five βCs on *F. oxysporum*.

βCs	Harmaine	Harmaline	Harmalol	Harmane	Harmol
IC_50_ (mg/mL)	0.143	0.331	0.798	0.050	0.161

## Data Availability

Not applicable.
